# Trichostatin A Rescues the Disrupted Imprinting Induced by Somatic Cell Nuclear Transfer in Pigs

**DOI:** 10.1371/journal.pone.0126607

**Published:** 2015-05-11

**Authors:** Yanjun Huan, Jiang Zhu, Bo Huang, Yanshuang Mu, Qingran Kong, Zhonghua Liu

**Affiliations:** 1 College of Life Science, Northeast Agricultural University, Harbin, Heilongjiang Province, China; 2 Dairy Cattle Research Center, Shandong Academy of Agricultural Sciences, Jinan, Shandong Province, China; Qingdao Agricultural University, CHINA

## Abstract

Imprinting disorders induced by somatic cell nuclear transfer (SCNT) usually lead to the abnormalities of cloned animals and low cloning efficiency. Histone deacetylase inhibitors have been shown to improve gene expression, genomic methylation reprogramming and the development of cloned embryos, however, the imprinting statuses in these treated embryos and during their subsequent development remain poorly studied. In this study, we investigated the dynamics of *H19/Igf2* methylation and transcription in porcine cloned embryos treated with trichostatin A (TSA), and examined *H19/Igf2* imprinting patterns in cloned fetuses and piglets. Our results showed that compared with the maintenance of *H19/Igf2* methylation in fertilized embryos, cloned embryos displayed aberrant *H19/Igf2* methylation and lower *H19/Igf2* transcripts. When TSA enhanced the development of cloned embryos, the disrupted *H19/Igf2* imprinting was largely rescued in these treated embryos, more similar to those detected in fertilized counterparts. Further studies displayed that TSA effectively rescued the disrupted imprinting of *H19/Igf2* in cloned fetuses and piglets, prevented the occurrence of cloned fetus and piglet abnormalities, and enhanced the full-term development of cloned embryos. In conclusion, our results demonstrated that aberrant imprinting induced by SCNT led to the abnormalities of cloned fetuses and piglets and low cloning efficiency, and TSA rescued the disrupted imprinting in cloned embryos, fetuses and piglets, and prevented the occurrence of cloned fetus and piglet abnormalities, thereby improving the development of cloned embryos. This study would have important implications in improving cloning efficiency and the health of cloned animals.

## Introduction

Though somatic cell nuclear transfer (SCNT) has been achieved in many species, overall cloning efficiency is still low, and the developmental abnormalities, including low birth rates, placental defect and large offspring syndrome, etc., frequently occur [1–3], limiting the application of SCNT technology in basic research, agriculture and medicine.

It is generally believed that the developmental abnormalities of cloned animals and low cloning efficiency are largely due to incomplete epigenetic reprogramming, among which, genomic imprinting is a valuable genetic marker for understanding epigenetic reprogramming and evaluating the developmental competence and normality of *in vitro* produced embryos [4–6].

Genomic imprinting is an epigenetic regulatory mechanism, showing a monoallelic, parental-specific expression, and crucial for embryo development [7]. In normal reproduction, genomic imprinting is established during gametogenesis and maintained throughout the subsequent development, however, during animal cloning, genomic imprinting is often disrupted, and abnormal expression of imprinted genes results in poor development of cloned embryos [6, 8]. And, in human assisted reproduction, the disruption of genomic imprinting usually leads to severe diseases, such as Beckwith Wiedemann syndrome and Angelman syndrome, influencing growth and development [4, 5, 9]. Increasing studies suggest that aberrant imprinting induced by SCNT could be the underlying cause of developmental abnormalities and low cloning efficiency [6, 8, 10].

To facilitate epigenetic reprogramming and cloning efficiency, epigenetic modification agents are usually applied, among which, histone deacetylase inhibitors could improve DNA damage repair, gene expression and genomic imprinting, etc., in cloned embryos, leading to the enhanced development of cloned embryos [11–13]. Our previous studies also report that TSA could enhance genomic methylation reprogramming and improve the development of cloned embryos [14, 15]. And more, TSA can normalize gene expression profile in cloned animals [16]. These studies suggest that TSA could improve the disrupted imprinting during animal cloning. However, the effects of TSA on the imprinting statuses of cloned embryos and animals remain unknown.

At present, *H19/Igf2*, representing genomic imprinting, is most studied in animals, and also well-known in pigs [6, 17]. Previous studies have shown that the expression patterns of *H19* and *Igf2*, controlled by the differentially methylated region (DMR) of *H19*, are maternal and paternal specific, respectively [7, 18, 19]. The DMR is preferentially methylated on the paternal allele, and the enhancer element prefers the paternal expression of *Igf2*. Otherwise, *H19* expression has a silencing effect on *Igf2* expression. This mechanism allows for the precise control of *H19* and *Igf2* expression. In this study, the DMR3 of *H19/Igf2* (widely accepted) was selected to investigate the effects of TSA on the imprinting statuses of cloned embryos, fetuses and piglets [17, 19]. Our results demonstrated that aberrant imprinting induced by SCNT led to the abnormalities of cloned fetuses and piglets, and TSA rescued the disrupted imprinting in cloned embryos, fetuses and piglets, and prevented the occurrence of cloned fetus and piglet abnormalities, thereby enhancing the full-term development of cloned embryos. This work provides a novel insight into the imprinting regulatory mechanism, and would have important implications in improving cloning efficiency and the health of cloned animals.

## Materials and Methods

Chemicals were purchased from Sigma Aldrich Corporation (St. Louis, MO, USA), and disposable and sterile plasticware was obtained from Nunclon (Roskilde, Denmark), unless otherwise stated.

All the treatments of piglets were approved by the Animal Care and Use Commission of Northeast Agriculture University, according to animal welfare laws, guidelines and policies. All pigs involved in this research were raised and bred according to the guideline of Animal Husbandry Department of Heilongjiang, China.

### Porcine adult fibroblast (PAFs) culture

PAFs were isolated from the ear of an American Large White boar. After removal of skin tissues and gristle, the remaining tissues were finely minced into pieces, digested with 0.25% trypsin-0.04% ethylenediaminetetraacetic acid solution (GIBCO), and dispersed in high glucose enriched Dulbecco’s modified Eagle’s medium (DMEM, GIBCO) containing 10% fetal bovine serum (FBS, GIBCO) and 1% penicillin-streptomycin (GIBCO). Then, the dispersed cells were centrifuged, resuspended and cultured in DMEM. Until confluence, PAFs were digested, centrifuged, resuspended in FBS containing 10% dimethyl sulfoxide and stored in liquid nitrogen until use. Prior to SCNT, PAFs were thawed, cultured and subsequently used in 3–5 passages.

### Oocyte collection and in vitro maturation (IVM)

Oocyte maturation has been described previously [20]. Briefly, porcine ovaries were collected from a slaughterhouse of Harbin Dazhong Roulian Food Co., Ltd., located in Harbin city, Heilongjiang province. Just after ovary exposure, they were placed in physiological saline with antibiotics at 37°C and transported to the laboratory. Follicles were aspirated, and follicular contents were washed with HEPES buffered Tyrode's lactate. Cumulus-oocyte complexes (COCs) were recovered, washed and cultured in maturation medium. After 42 h, COCs were vortexed in hyaluronidase to remove cumulus cells. Only oocytes with a visible polar body, regular morphology and homogenous cytoplasm were used.

### In vitro fertilization (IVF) and SCNT embryo culture, treatment and collection

The procedures for porcine IVF and SCNT have been described in one of our previous reports [21]. Briefly, for IVF, the semen was incubated, resuspended and washed in DPBS supplemented with 0.1% (w/v) BSA. The spermatozoa were diluted with modified Tris-buffered medium (mTBM) to the appropriate concentration. Matured oocytes were washed in mTBM, transferred into fertilization medium and coincubated with spermatozoa. Then, the embryos were washed and cultured in porcine zygote medium-3 (PZM-3) for subsequent development. For SCNT, matured oocytes and PAFs were placed in manipulation medium. After enucleation, donor cells were placed into the perivitelline space. Fusion and activation of the cell-cytoplast complexes were induced by electroporation, and the fusion rate was confirmed by microscopic examination. Reconstructed embryos were cultured in PZM-3 supplemented with 40 nM (optimized) TSA (NT-TSA) for 24 h [14, 15], washed and transferred into PZM-3 for subsequent development. The cleavage and blastocyst rates were evaluated at 48 h and 156 h, respectively. For embryo collection, the 1-cell, 2-cell, 4-cell, 8-cell and blastocyst stage embryos in the IVF, NT-CON (cloned) and NT-TSA groups were collected at 6 h, 24 h, 48 h, 72 h and 156 h, respectively.

### Embryo transfer, pregnancy diagnosis and birth

Embryo transfer has been described [22]. About 200 embryos per a recipient were kept in manipulation medium, transported in a portable incubator, and loaded into a sterilized straw before transfer. A recipient of natural oestrus synchronization was anaesthetized, and the oviduct was exposed by laparotomy. Then, the embryo-loaded straw was inserted into the oviduct, and embryos were injected. Pregnancy diagnosis was performed by ultrasonography every 30 days and fetuses were obtained at the 35th day after transfer. After birth, the survival rates and the weight of piglets were examined.

### Bisulfite sequencing

Bisulfite sequencing has been reported [15]. Briefly, pooled samples were treated with sodium bisulfite to convert all unmethylated cytosine to uracil using an EZ DNA Methylation-Direct Kit (Zymo Research) according to the manufacturer’s protocol. For semen, the sperm was collected by centrifugation, washed and incubated in SMB solution supplemented with 40 mM dithiothreitol and 0.3 mg ml^-1^ Proteinase K at 56°C for 1 h. For PAFs, fetus and piglet tongue tissues, a Universal Genomic DNA Extraction Kit (TaKaRa) was used to extract genomic DNA. For samples of 200 MII oocytes and 200 1-cell, 100 2-cell, 50 4-cell, 30 8-cell and 10 blastocyst stage pooled zona pellucida-removed embryos, digestion was performed in M-Digestion Buffer plus with Proteinase K at 50°C for 20 min. After treatment, CT (cytosine to thymine) conversion reagents were added to all the samples at 98°C for 10 min and 64°C for 2.5 h. Then, the samples were desalted, purified and diluted with M-Elution Buffer. Subsequently, nested PCR was carried out to amplify DMR3 of *H19/Igf2* using previously reported primers as described in S1 Table [19] and Hot Start Taq Polymerase (TaKaRa) with a profile of 94°C for 5 min, 40 cycles of 94°C for 30 sec, 55°C for 30 sec and 72°C for 1 min, followed by 72°C for 10 min. Products from the first amplification reaction were used in the second PCR reaction, and the optimal annealing temperature of inner primers was 50°C. Then, the amplified products were verified by electrophoresis and purified using an Agarose Gel DNA Purification Kit (TaKaRa), and the purified fragments were cloned into a pMD18-T Vector (TaKaRa) and subjected to sequence analysis.

## Quantitative Real Time PCR

Measurement of gene expression with quantitative real time PCR has been applied in our previous studies [20, 21]. Briefly, total RNA was extracted from 50 embryos at each stage and fetus and piglet tongue tissues using an RNeasy Mini Kit (Qiagen) according to the manufacturer’s instruction. Reverse transcription was performed using a PrimeScript RT Reagent Kit (TaKaRa) with the following parameters: 37°C for 15 min and 85°C for 5 sec, and the cDNA was stored at -20°C until use. For quantitative real time PCR, reactions were performed in 96-well optical reaction plates using SYBR Premix ExTaq II (TaKaRa) and a 7500 Real-Time PCR System (Applied Biosystems) with the following conditions: 95°C for 30 sec, followed by 40 two-step cycles of 95°C for 5 sec and 60°C for 34 sec and finally a dissociation stage consisting of 95°C for 15 sec, 60°C for 1 min and 95°C for 15 sec. For every sample, the cycle threshold (CT) values were obtained from three replicates. The primers used for amplification of target and internal reference genes are presented in S1 Table. The relative expression levels of target genes were analyzed using the 2^−ΔΔCT^ method.

### Statistical analysis

Differences in data (mean ± SEM) were analyzed with SPSS statistical software. Statistical analysis of data regarding embryo development was performed using t-text. The data for gene expression and DNA methylation were analyzed with one-way ANOVA. For all analyses, differences were considered to be statistically significant when P<0.05.

## Results

### Aberrant imprinting of *H19/Igf2* in porcine cloned embryos

**Fig 1:**
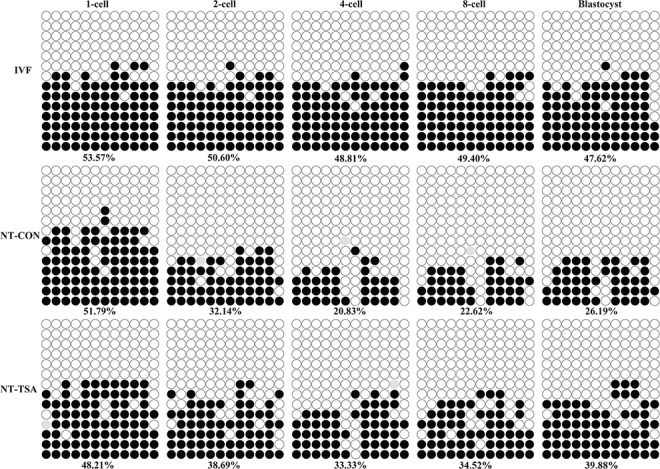
H19/Igf2 methylation statuses in early embryos. The methylation statuses of H19/Igf2 at 1-cell, 2-cell, 4-cell, 8-cell and blastocyst stages of IVF, NT-CON and NT-TSA embryos were examined. Cloned embryos displayed aberrant imprinting of H19/Igf2, while TSA rescued the disrupted imprinting in cloned embryos. Black or white circles indicate methylated or unmethylated CpG sites, respectively, and gray circles represent mutated and/or single nucleotide polymorphism (SNP) variation at certain CpG sites.

The methylation statuses of *H19/Igf2* in sperm and MII oocytes were examined, showing hypermethylation in sperm and hypomethylation in MII oocytes (S1 Fig). After fertilization, *H19/Igf2* methylation did not obviously change at the 1-cell stage in comparison with the mean methylation level of sperm and oocytes (53.57% vs 49.11%), and was maintained from the 1-cell to blastocyst stage (Fig 1), indicating that IVF embryos maintained imprinting.

**Fig 2:**
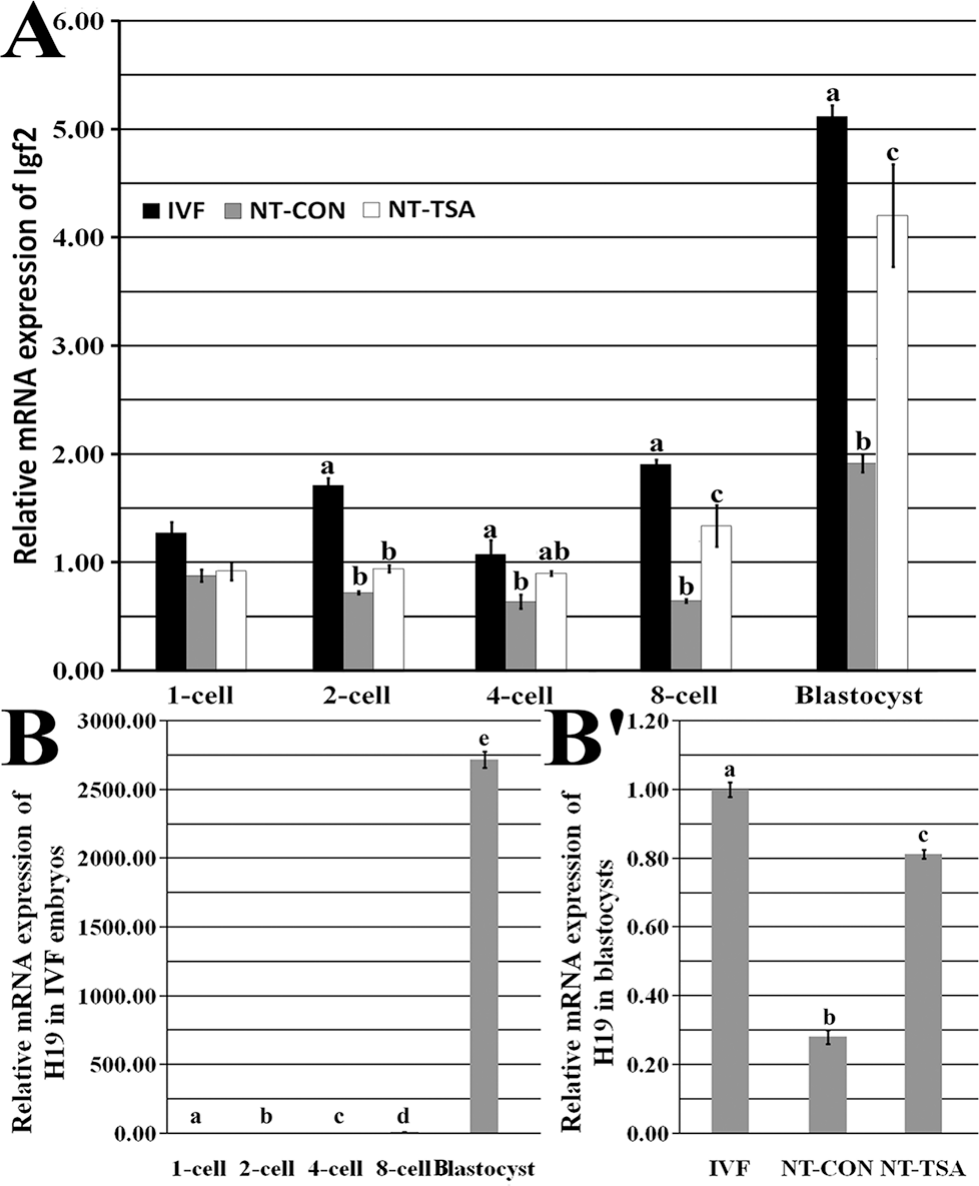
Relative H19/Igf2 transcripts in early embryos. A, relative Igf2 transcripts at 1-cell, 2-cell, 4-cell, 8-cell and blastocyst stages of IVF, NT-CON and NT-TSA embryos, B, H19 transcripts in IVF embryos, and B', H19 transcripts in blastocysts of IVF, NT-CON and NT-TSA embryos. TSA improved the expression levels of H19/Igf2 in cloned embryos. The transcript abundance in MII stage oocytes (A and B) or IVF blastocysts (B') was considered to be the control. The data were expressed as mean ± SEM. ^a-e^Values with different superscripts differ significantly (P<0.05).

After SCNT (Fig 1 and S1 Fig), though no significant differences of *H19/Igf2* methylation were observed between donor cells and 1-cell stage embryos, cloned embryos displayed a gradual and obvious demethylation from the 1-cell to 4-cell stage and a slight remethylation from the 4-cell to blastocyst stage, and the methylation levels of *H19/Igf2* were obviously lower than those in IVF embryos from the 2-cell to blastocyst stage, suggesting that aberrant imprinting was present in cloned embryos. As for the expression of *H19/Igf2*, compared with IVF embryos, cloned embryos displayed significantly lower transcripts of *Igf2* from the 2-cell to blastocyst stage and *H19* in blastocysts (Fig 2, P<0.05). These results suggested that SCNT disrupted the imprinting of *H19/Igf2* in cloned embryos.

### TSA rescued the disrupted imprinting in porcine cloned embryos

When TSA was applied to treat cloned embryos, the transcripts of histone acetylation (*Hat1* and *Hdac1*) and DNA methylation (*Dnmt1* and *Dnmt3a*) related genes were significantly improved (S2 Fig, P<0.05), and the development of cloned embryos were also significantly enhanced (Table 1 and S3 Fig, P<0.05). Then, we analyzed the imprinting statuses of *H19/Igf2* in TSA-treated cloned embryos (Fig 1). The results of *H19/Igf2* methylation demonstrated that 1-cell to 4-cell stage embryos underwent a partial demethylation and 4-cell to blastocyst stage embryos took on a restored remethylation. And, 2-cell to blastocyst stage embryos almost maintained a certain imprinting in the NT-TSA group. When compared with the NT-CON group, the NT-TSA group displayed higher methylation levels of *H19/Igf2* from 2-cell to blastocyst stage embryos, much closer to the IVF group. Furthermore, the transcripts of *Igf2* at the 8-cell and blastocyst stages and *H19* at the blastocyst stage in the NT-TSA group were significantly higher than those in the NT-CON group, though still significantly lower in comparison with the IVF group (Fig 2, P<0.05). Overall, TSA largely rescued the disrupted imprinting of *H19/Igf2* in cloned embryos.

**Table 1:** *In vitro* development of cloned embryos treated with TSA.

Groups	No. embryos (Rep.)	No. embryos cleaved (% ± SEM)	No. blastocysts (% ± SEM)
**NT-CON**	242 (5)	209 (85.79 ± 0.95)	50 (20.50 ± 0.70)^a^
**NT-TSA**	238 (5)	210 (88.82 ± 1.12)	118 (50.71 ± 2.21)^b^

40 nM TSA enhanced the development of cloned embryos.

^a-b^Values in the same column with different superscripts differ significantly (P<0.05).

### TSA reduced the abnormalities of cloned fetuses and piglets

After TSA-treated cloned embryos were transferred to surrogates, higher rates of pregnancy, offspring and alive offspring were observed in the NT-TSA group in comparison with those in the NT-CON group (Table 2).

**Table 2:** *In vivo* development of cloned embryos treated with TSA.

Group	No. surrogate	No. pregnancy (%)^a^	No. delivery	No. offspring (mean)^b^	No. offspring alive (%)^c^
**NT-CON**	10	4 (40.00)	3	8 (2.67)	3 (37.50)
**NT-TSA**	5	3 (60.00)	2	7 (3.50)	6 (85.71)

After treatment with TSA, *in vivo* development of cloned embryos was improved.

^a^The pregnant rate was based on the surrogate number and one pregnant surrogate in each group was used for fetus examination.

^b^The number of offspring was adjusted for the delivery number.

^c^The number of alive offspring was based on the offspring number.

**Fig 3:**
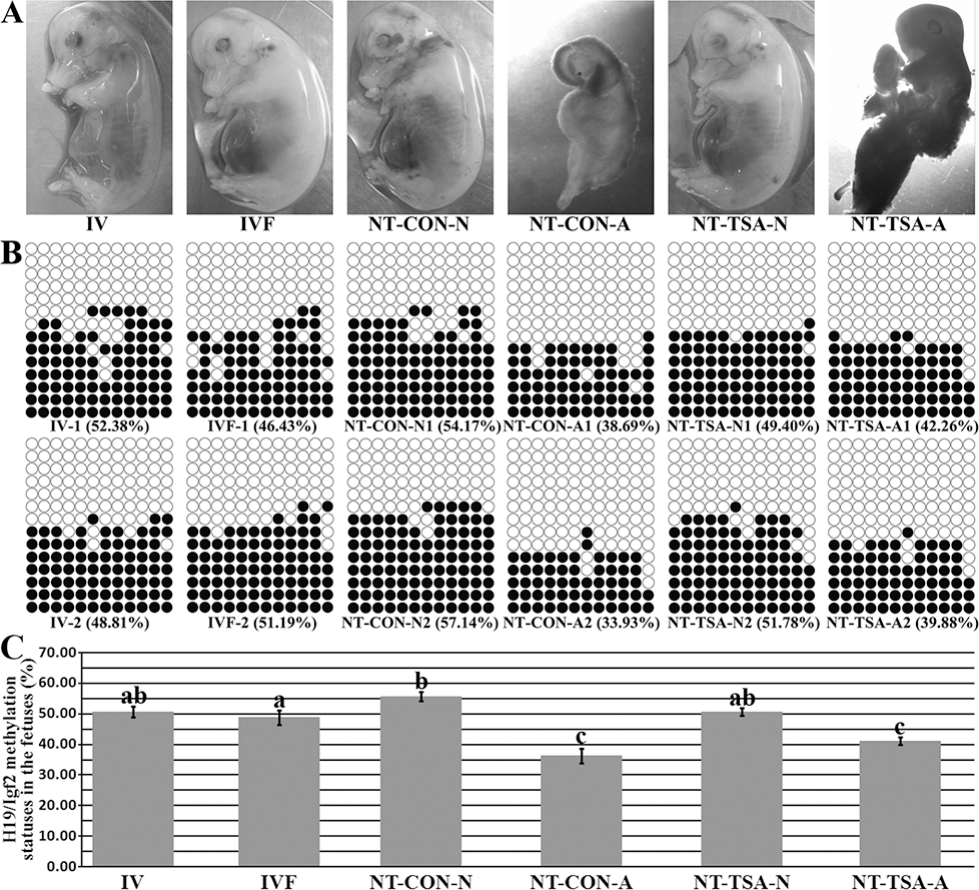
Methylation statuses of H19/Igf2 in 35-day fetuses. A, the morphologies of 35-day fetuses, B and C, the corresponding methylation statuses of H19/Igf2 in 35-day fetuses. IV, the in vivo produced fetuses, IVF, the IVF fetuses, NT-CON-N, the normal cloned fetuses in the NT-CON group, NT-CON-A, the abnormal cloned fetuses in the NT-CON group, NT-TSA-N, the normal cloned fetuses in the NT-TSA group, and NT-TSA-A, the abnormal cloned fetuses in the NT-TSA group. TSA prevented the H19/Igf2 imprinting disruption and morphological abnormality of cloned fetuses. Black or white circles indicate methylated or unmethylated CpG sites. The data were expressed as mean ± SEM. ^a-c^Values with different superscripts differ significantly (P<0.05).

**Fig 4:**
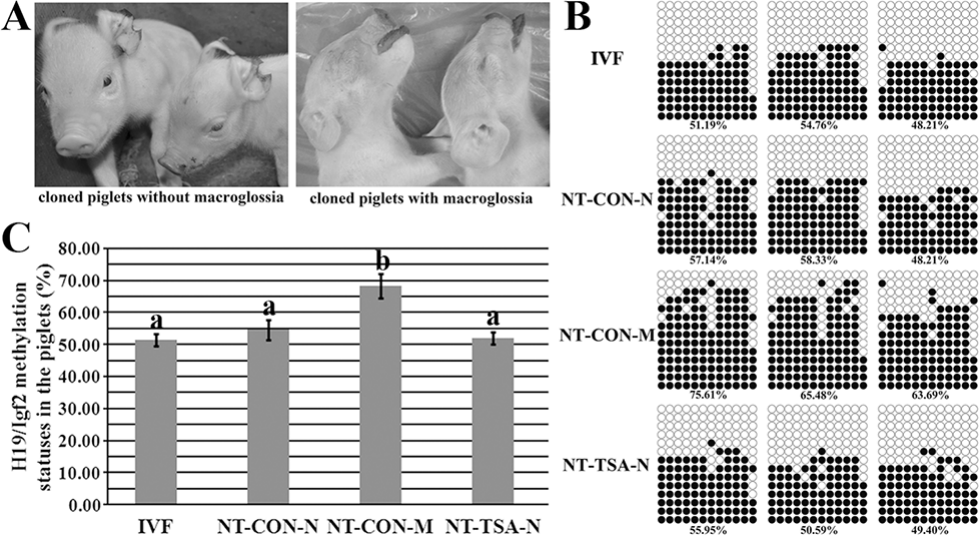
H19/Igf2 methylation statuses in piglets. A, cloned piglets without or with macroglossia, B and C, methylation statuses of H19/Igf2 in cloned piglets. IVF, the IVF piglets, NT-CON-N, normal cloned piglets in the NT-CON group, NT-CON-M, cloned piglets with macroglossia in the NT-CON group, and NT-TSA-N, normal cloned piglets in the NT-TSA group. TSA prevented the disrupted imprinting of *H19/Igf2* and macroglossia occurrence of cloned piglets. Black or white circles indicate methylated or unmethylated CpG sites, respectively. The data were expressed as mean ± SEM. ^a-b^Values with different superscripts differ significantly (P<0.05).

During *in vivo* development of cloned embryos, the morphologies of cloned fetuses and piglets were examined. For 35-day cloned fetuses (Fig 3), there were 4 abnormal fetuses of 6 cloned fetuses in the NT-CON group, while only 2 of 5 cloned fetuses in the NT-TSA group were abnormal. Interestingly, the sizes of abnormal cloned fetuses were obviously smaller than those of normal fetuses, including 2 fetuses in the NT-CON group, 3 fetuses in the NT-TSA group, 7 fetuses in the IVF group and 6 fetuses in the IV group. When birth (Fig 4), 5of 8 cloned piglets died of macroglossia in the NT-CON group (namely the NT-CON-M group), while macroglossia did not occur (that is the NT-TSA-N group), though one cloned piglet died (as the NT-TSA-D group) in the NT-TSA group. Thus, TSA effectively reduced the abnormalities of cloned fetuses and piglets.

### TSA prevented the disrupted imprinting of *H19/Igf2* in cloned fetuses and piglets

**Fig 5:**
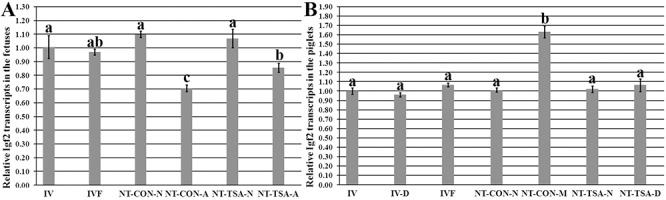
Relative Igf2 transcripts in fetuses and piglets. A, Igf2 transcripts in fetuses, and B, Igf2 expression levels in piglets. IV, the in vivo produced fetuses or piglets, IV-D, the in vivo produced dead piglets, IVF, the IVF fetuses or piglets, NT-CON-N, the normal cloned fetuses or piglets in the NT-CON group, NT-CON-A, the abnormal cloned fetuses in the NT-CON group, NT-CON-M, cloned piglets with macroglossia in the NT-CON group, NT-TSA-N, the normal cloned fetuses or piglets in the NT-TSA group, NT-TSA-A, the abnormal cloned fetuses in the NT-TSA group, and NT-TSA-D, the cloned dead piglets in the NT-TSA group. TSA improved the expression of Igf2 in cloned fetuses and piglets. The transcript abundance in the in vivo produced fetuses (A) or piglets (B) was considered to be the control. The data were expressed as mean ± SEM. ^a-c^Values with different superscripts differ significantly (P<0.05).

Then, the imprinting statuses of *H19/Igf2* in cloned fetuses and piglets were examined. For 35-day cloned fetuses in the NT-CON group (Fig 3), the abnormal cloned fetuses displayed significantly lower methylation levels of *H19/Igf2* in comparison with the IV or IVF fetuses (P<0.05), while the normal cloned fetuses showed significantly (P<0.05) higher *H19/Igf2* methylation levels than the IVF fetuses and no significant differences from IV fetuses. And, *Igf2* transcripts in the abnormal cloned fetuses were significantly lower than the normal cloned fetuses, IVF and IV fetuses (Fig 5, P<0.05). In the NT-TSA group, there were no significant differences of the methylation and expression levels of *H19/Igf2* between the normal cloned fetuses and IV or IVF fetuses, and the abnormal cloned fetuses also displayed higher *H19/Igf2* methylation and expression levels than their counterparts in the NT-CON group, though still significantly lower than the normal cloned, IV and IVF fetuses. Thus, TSA prevented the imprinting disruption of cloned fetuses.

When birth, further studies displayed that the methylation and expression levels of *H19/Igf2* in the NT-CON-M group were significantly higher than those in the NT-CON-N (cloned piglets without macroglossia), NT-TSA-N and IVF groups (Figs 4 and 5, P<0.05). After TSA treatment, even in the NT-TSA-D group, the methylation and expression levels of *H19/Igf2* were still similar to those of the IV (live or dead), IVF and normal cloned piglets (Figs 4 and 5 and S4 Fig). Overall, TSA effectively reduced the imprinting disruption in cloned fetuses and piglets.

## Discussion

Genomic imprinting is essential for normal development of early embryos [6]. In this study, we demonstrated that the imprinting disruption induced by SCNT led to cloned fetus abnormalities and the occurrence of macroglossia in cloned piglets, and TSA could rescue the disrupted imprinting, prevent the occurrence of abnormalities in cloned fetuses and piglets, and enhance the development of cloned embryos.

Genomic imprinting is an epigenetic mechanism that ensures parental monoallelic expression, and crucial for embryo development, fetus growth and postnatal behaviors [7], and the *H19/Igf2* locus (DMR3) can represent genomic imprinting to be a valuable genetic marker for evaluating the developmental competence and normality of *in vitro* produced embryos [17, 19]. Here, the DMR3 of *H19/Igf2* was chosen to evaluate the reprogramming of genomic imprinting during pig cloning.

Naturally, genomic imprinting is established during gametogenesis and maintained through the subsequent development, however, SCNT bypasses this step and destroys genomic imprinting [6, 8, 23]. In this study, porcine fertilized embryos maintained the imprinting of *H19/Igf2*, coinciding with previous reports [19, 21], whereas, aberrant imprinting was observed in cloned embryos, suggesting that the key molecules for genomic imprinting maintenance may be lost during SCNT [21]. Moreover, increasing studies also display that genomic imprinting is severely compromised in SCNT embryos, probably leading to the low developmental competence of cloned embryos [21, 23, 24].

Our previous studies have demonstrated that histone deacetylase inhibitors can improve genomic methylation reprogramming and the development of cloned embryos [14, 15], and recent reports also reveal that histone modifications could regulate genomic imprinting [7, 11]. Accordingly, the improvement of histone acetylation modification largely rescued genomic imprinting in porcine cloned embryos, closer to fertilized counterparts, and this result is similar to one previous report [11]. As for the reason, it is possible that the improvement of histone acetylation modification opens the chromatin structure and makes the regulatory molecules close to the imprinting region and maintain genomic imprinting [7, 25]. Moreover, *H19/Igf2* transcripts were obviously upregulated in cloned blastocysts after TSA treatment, possibly benefit for the post-implantation development [24]. Thus, TSA has the ability to regulate genomic imprinting. As which molecules rescue the disrupted imprinting in cloned embryos after TSA treatment [26], further studies are needed.

The improvement of genomic imprinting in early cloned embryos would enhance their subsequent development or viability [6, 11]. The pregnancy and birth rates and the abnormal proportions of cloned fetuses and piglets in the NT-TSA group proved this view, and suggest that imprinting maintenance during early development is required for the implantation and subsequent development of cloned embryos. For the fetuses, along with the reduced sizes, low methylation and expression levels of *H19/Igf2* were usually observed in abnormal cloned fetuses. This may be due to that the downregulated methylation levels of *H19/Igf2* led to the reduced *Igf2* expression, further resulting in the small and abnormal fetuses [18, 27]. Interestingly, the abnormal fetuses in the NT-CON group displayed even smaller sizes and lower methylation and expression levels of *H19/Igf2* compared with those in the NT-TSA group. Thus, to some extent, the imprinting improvement by TSA treatment promotes the development of cloned fetuses.

When birth, most cloned piglets died of macroglossia in the NT-CON group, and the weight was higher than those of the live cloned or fertilized piglets. Notably, these cloned piglets with macroglossia were accompanied with the high methylation and expression levels of *H19/Igf2*, similar to Beckwith Wiedemann syndrome in human assisted reproduction [28, 29]. Previous studies also display that genomic imprinting is aberrant in cloned piglets with macroglossia [30, 31]. Thus, the occurrence of macroglossia in cloned piglets could be attributed to the disrupted imprinting. As how the methylation degree of *H19/Igf2* DMR3 is upregulated, further leading to high *Igf2* expression and piglet abnormality [18, 27], it may be due to the disrupted nutritional transport pathways [31], and more studies are still needed. Encouragingly, no macroglossia occurred in the NT-TSA group, even the only one deceased cloned piglet, and the imprinting of *H19/Igf2* were normal in the NT-TSA group, suggesting that TSA has a correcting effect on genomic imprinting. And more, TSA has been reported to normalize gene expression in cloned animals [16]. Thus, TSA could reduce the abnormalities of cloned fetuses and piglets by regulating genomic imprinting, bringing good news to animal cloning and human assisted reproduction. Certainly, during animal cloning, multiple imprinted genes are involved and numerous molecules could regulate genomic imprinting [6, 7, 31, 32], thus, more information is needed to clarify the role of TSA in the imprinting regulatory mechanism.

In conclusion, our results demonstrated that aberrant imprinting induced by SCNT led to the abnormalities of cloned fetuses and piglets, and TSA rescued the disrupted imprinting in cloned embryos, fetuses and piglets, and prevented the occurrence of cloned fetus and piglet abnormalities, thereby enhancing the development of cloned embryos.

## Supporting Information

S1 Fig H19/Igf2 methylation statuses. A, hypermethylation in sperm, B, hypomethylation in MII stage oocytes, and C, moderate methylation in PAFs. Black or white circles represent methylated or unmethylated CpG sites.
(TIF)

S2 Fig Relative transcripts of histone acetylation and DNA methylation related genes in early embryos. The expression patterns of Hat1 (A), Hdac1 (B), Dnmt1 (C) and Dnmt3a (D) at the 1-cell, 2-cell, 4-cell, 8-cell and blastocyst stages of IVF, NT-CON and NT-TSA embryos. In comparison with IVF embryos, cloned embryos displayed the disrupted expression patterns of Hat1, Hdac1, Dnmt1 and Dnmt3a, while these gene expression profiles in NT-TSA embryos were almost normal. The transcript abundance in MII stage oocytes was considered to be the control. The data were expressed as mean ± SEM. ^a-b^Values with different superscripts differ significantly (P<0.05).
(TIF)

S3 Fig Blastocysts of cloned embryos. A, blastocysts (×40) derived from cloned embryos, and B, blastocysts (×40) derived from cloned embryos treated with 40 nM TSA.
(TIF)

S4 Fig Methylation statuses of H19/Igf2 in piglets. IV-D, the dead piglet derived from the IV group, and NT-TSA-D, the dead piglet derived from the NT-TSA group. The IV, IV-D and NT-TSA-D piglets displayed nearly normal H19/Igf2 methylation. Black and white circles represent methylated and unmethylated CpG sites.
(TIF)

S1 Table Detail of primers for bisulfite sequencing and quantitative real time PCR. The primer sequence, amplified length and gene accession number for bisulfite sequencing and quantitative real time PCR.
(PDF)
