# Supplementary figures and images for: Trichostatin A Rescues the Disrupted Imprinting Induced by Somatic Cell Nuclear Transfer in Pigs
Source: PLoS One. 2015 May 11;10(5):e0126607. doi: 10.1371/journal.pone.0126607 (PMC4427324; doi:10.1371/journal.pone.0126607)

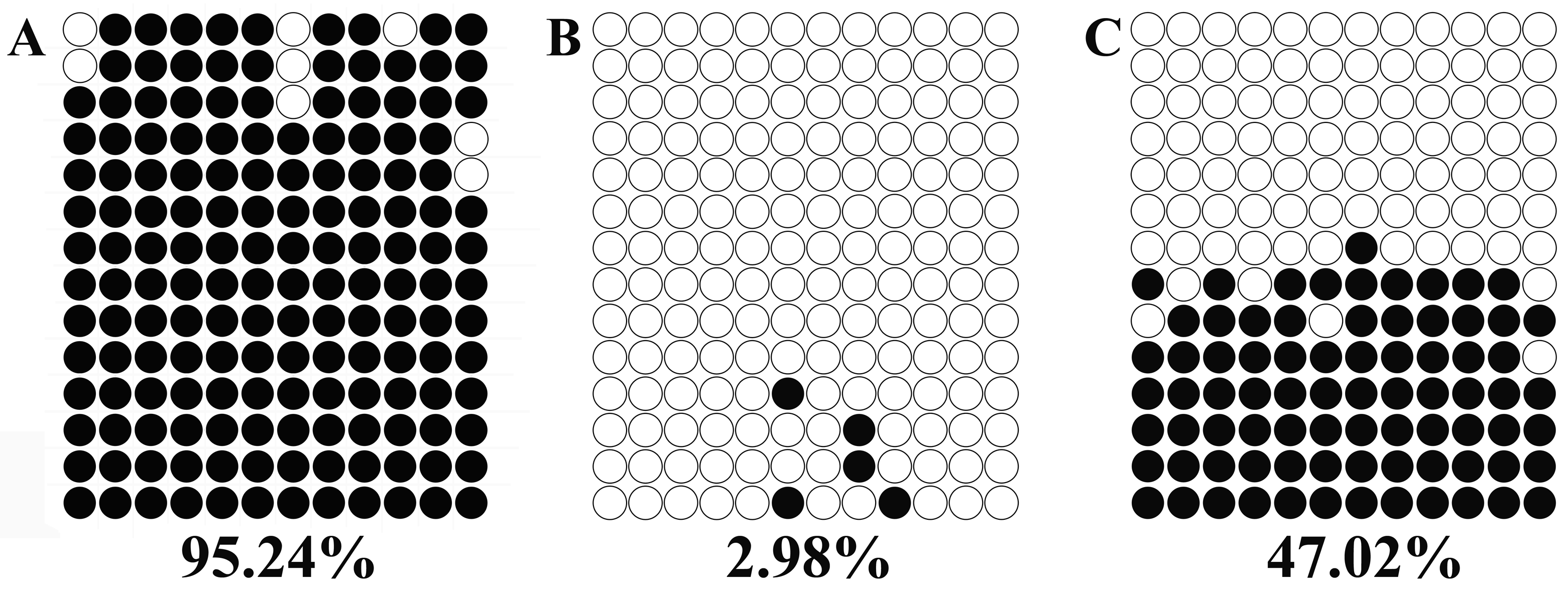

Supplement: S1 Fig — A, hypermethylation in sperm, B, hypomethylation in MII stage oocytes, and C, moderate methylation in PAFs. Black or white circles represent methylated or unmethylated CpG sites. (TIF) [file pone.0126607.s001.tif]

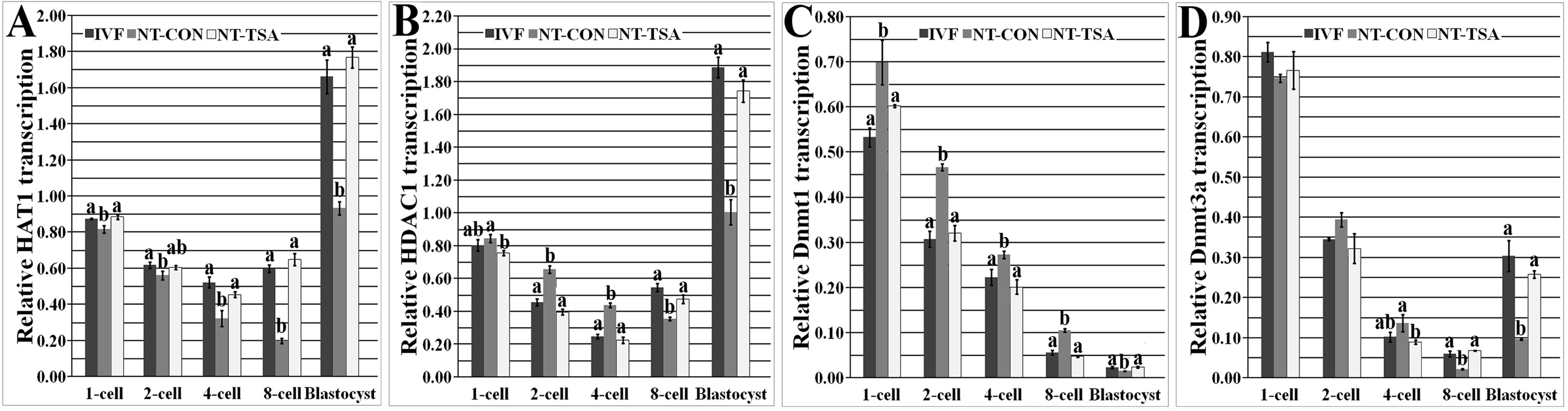

Supplement: S2 Fig — The expression patterns of Hat1 (A), Hdac1 (B), Dnmt1 (C) and Dnmt3a (D) at the 1-cell, 2-cell, 4-cell, 8-cell and blastocyst stages of IVF, NT-CON and NT-TSA embryos. In comparison with IVF embryos, cloned embryos displayed the disrupted expression patterns of Hat1, Hdac1, Dnmt1 and Dnmt3a, while these gene expression profiles in NT-TSA embryos were almost normal. The transcript abundance in MII stage oocytes was considered to be the control. The data were expressed as mean ± SEM. a-bValues with different superscripts differ significantly (P<0.05). (TIF) [file pone.0126607.s002.tif]

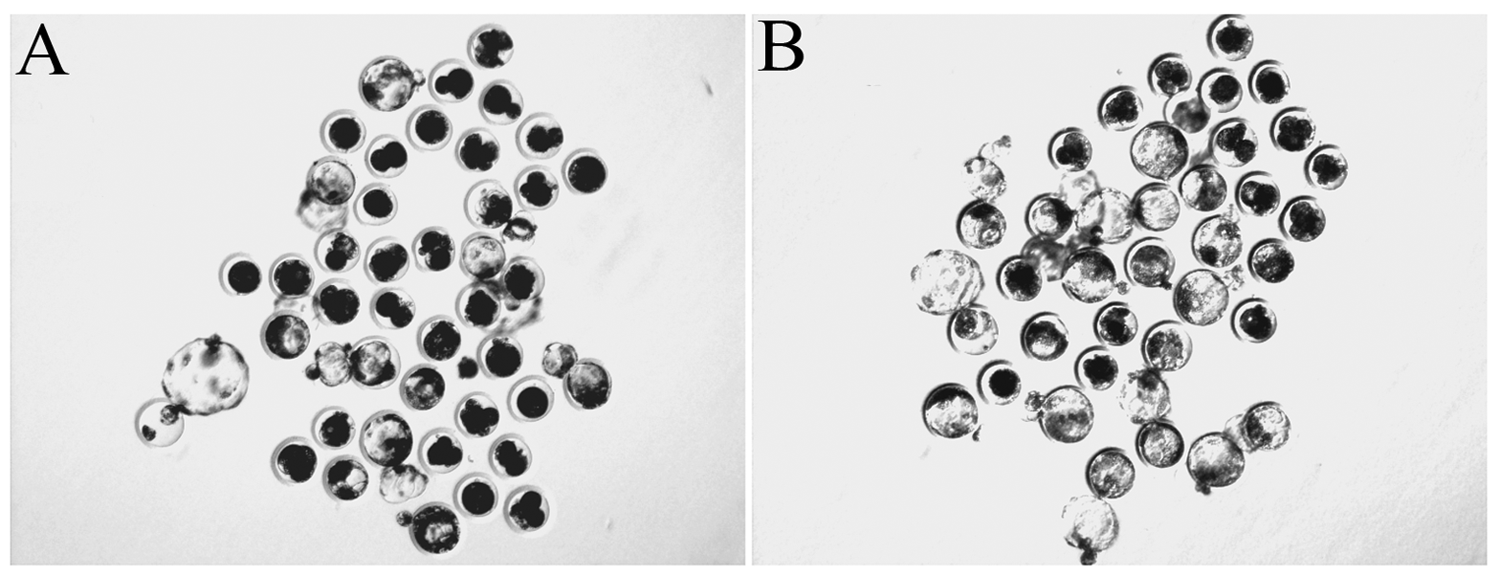

Supplement: S3 Fig — A, blastocysts (×40) derived from cloned embryos, and B, blastocysts (×40) derived from cloned embryos treated with 40 nM TSA. (TIF) [file pone.0126607.s003.tif]

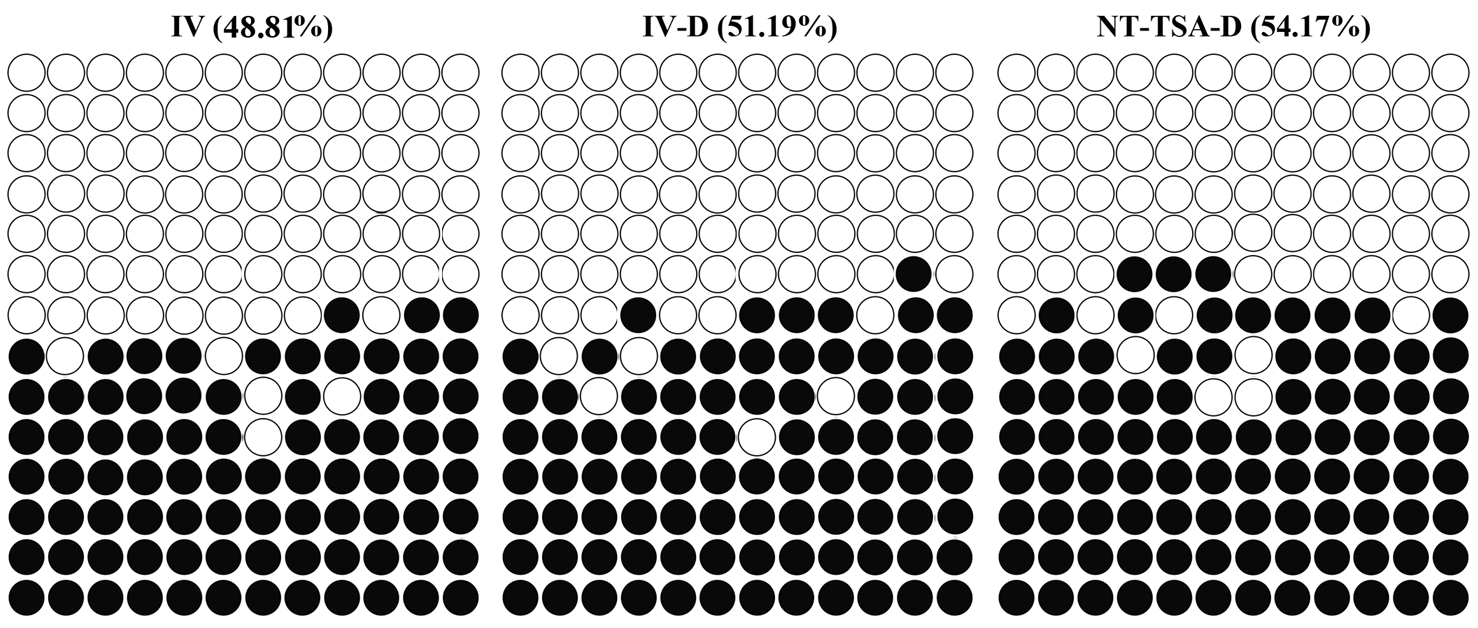

Supplement: S4 Fig — IV-D, the dead piglet derived from the IV group, and NT-TSA-D, the dead piglet derived from the NT-TSA group. The IV, IV-D and NT-TSA-D piglets displayed nearly normal H19/Igf2 methylation. Black and white circles represent methylated and unmethylated CpG sites. (TIF) [file pone.0126607.s004.tif]
