# Supplementary material for: Trichostatin A Rescues the Disrupted Imprinting Induced by Somatic Cell Nuclear Transfer in Pigs
Source: PLoS One. 2015 May 11;10(5):e0126607. doi: 10.1371/journal.pone.0126607 (PMC4427324; doi:10.1371/journal.pone.0126607)
Supplement: S1 Table — The primer sequence, amplified length and gene accession number for bisulfite sequencing and quantitative real time PCR. (PDF) [file pone.0126607.s005.pdf]

S1 Table. Detail of primers for bisulfite sequencing and quantitative real time PCR.

Gene	Primer sequence (5'-3')	Length (bp)	Accession number
**Bisulfite sequencing analysis**			
**H19/Igf2**	Outer<br>F:GGTTTTAGGGGGATATTTTTT<br>R:TTAAAAAAACATTACTTCCATATAC	384	AY044827
	Inner<br>F:GATTTTTAGGTTTGTTATTATTT<br>R:CAAATATTCAATAAAAAAACCC	208	
**Gene expression analysis**			
**Igf2**	F:CCGTGGCATCGTGGAAGAGTG<br>R:TCCAGGTGTCATAGCGGAAGAAC	170	NM_213883
**H19**	F: TCAAACGACAAGAGATGGTGCTA<br>R: CAAGTAGTGTAGTGGCTCCAGAATG	118	AY044827
**Hat1**	F: GACTGTGTGGAGGCAGATGATGTA<br>R:GAGAACCGAGTATGTATGGAGTAAGG	150	XM_005671969
**Hdac1**	F: GCTGGCAAAGGCAAGTATTATG<br>R: CACACTGTAAGACGACCGCAC	139	XM_005665200
**Dnmt1**	F:GCGTCTTGCAGGCTGGTCAGTA<br>R:CTTCTTATCATCGACCACGACGCT	152	NM_001032355
**Dnmt3a**	F:ATGTGGTTCGGAGACGGCAAGT<br>R:GCTCTCGTCGTTGTCATGGCA	195	NM_001097437
**β2m**	F:CTTTCTACCTTCTGGTCCACACTG	116	NM_213978

	R:GTGGTCTCGATCCCACTTAACTATC		
**18s**	F:AATCTCGGGTGGCTGAACGC<br>R:CCGTTCTTAGTTGGTGGAGCGAT	143	NR_002170
